# Prostatic Abscess Caused by *Klebsiella pneumoniae*: A 6-Year Single-Center Study

**DOI:** 10.3390/jcm11092521

**Published:** 2022-04-29

**Authors:** Joo-Hee Hwang, Jeong-Hwan Hwang, Seung Yeob Lee, Jaehyeon Lee

**Affiliations:** 1 Department of Internal Medicine, Jeonbuk National University Medical School and Hospital, Jeonju 54907, Korea; j.hwang@jbnu.ac.kr (J.-H.H.); mdhwang@jbnu.ac.kr (J.-H.H.); 2 Research Institute of Clinical Medicine of Jeonbuk National University—Biomedical Research Institute of Jeonbuk National University Hospital, Jeonju 54907, Korea; seungyeoblee@jbnu.ac.kr; 3 Department of Laboratory Medicine, Jeonbuk National University Medical School and Hospital, Jeonju 54907, Korea

**Keywords:** prostatic abscess, *Klebsiella pneumoniae*, hypervirulence

## Abstract

Hypervirulent *Klebsiella pneumoniae* (hvKp) is an important strain that can cause multiple organ infections. Although hvKp infection cases are increasing, there is limited information on the prostatic abscesses caused by *K. pneumoniae*. Furthermore, the clinical significance of hvKp associated with K1 or K2 capsular types or virulence genes in prostatic abscesses remains unclear. Therefore, we aimed to elucidate the clinical and microbiological characteristics of prostatic abscesses caused by *K. pneumoniae* in relation to various virulence genes. A retrospective study was performed at a 1200-bed tertiary hospital between January 2014 and December 2019. Patients diagnosed with prostatic abscesses with *K. pneumoniae* isolated from blood, urine, pus, or tissue cultures were enrolled in this study. Our results demonstrate that 30.3% (10/33) of the prostatic abscesses were caused by *K. pneumoniae*. All strains isolated from patients with prostatic abscesses due to *K. pneumoniae* were the K1 capsular type, and eight patients (80.0%) carried *rmpA* and *iutA* genes that identified hvKp. These findings suggest that hvKp is an important pathogen in prostatic abscesses. Therefore, when treating patients with *K. pneumoniae* prostatic abscesses, attention should be paid to the characteristics of hvKp, such as bacteremia, multiorgan abscess formation, and metastatic spread.

## 1. Introduction

The incidence of prostatic abscesses has declined markedly since the advent of broad-spectrum antibiotics [1]. Making the clinical diagnosis of prostatic abscesses is difficult due to the lack of pathognomonic symptoms or signs. However, advanced diagnostic tools, such as ultrasonography, computed tomography, or magnetic resonance imaging, allow the rapid and accurate diagnosis of prostatic abscesses [2,3,4]. Despite these advances, which reduce mortality by 3–16% in healthy patients with prostatic abscesses, the diagnosis and appropriate treatment of prostatic abscesses remain a challenge for physicians [5].

Before the widespread use of antibiotics, *Neisseria gonorrhea* was the most frequent causative pathogen of prostatic abscesses, accounting for 75% of cases among immunocompetent patients [1,6]. However, the introduction of antibiotics has resulted in a predominance of Gram-negative bacteria, particularly *Escherichia coli* (60–80%), as the most common microorganism causing prostatic abscesses [1,7]. Furthermore, *Klebsiella pneumoniae* and *Pseudomonas aeruginosa* are significant pathogens [8]. The prevalence of prostatic abscesses caused by *K. pneumoniae* varies with geographic area. In southern Taiwan, *K. pneumoniae* is the leading causative pathogen of prostatic abscesses [9], whereas in South Korea, *K. pneumoniae* (17.3%) is the second most common microorganism, following *E. coli* (40.4%) [10].

*K. pneumoniae* is an increasingly important microorganism capable of causing severe organ damage. Two types of *K. pneumoniae*, “classic” *K. pneumoniae* (cKp) and hypervirulent *K. pneumoniae* (hvKp), are currently in circulation. cKp is an opportunistic pathogen that causes infections in immunocompromised patients, primarily in healthcare settings [11]. Although the characteristics of hvKp and their differences from cKp are less well-known, hvKp is best understood as a virulent pathogen. The characteristic features of hvKp infection are its ability to infect a relatively healthy population of any age and its tendency to cause multiple infections at various anatomical sites [11]. Although the role of capsular types in virulence is not yet clearly defined, capsular types K1, K2, K5, K20, K54, and K57 are known to be correlated with hvKp strains [12]. Among these capsular types, K1 and K2 are not the most virulent but the most common capsular types associated with hvKp [13,14]. In addition, a recent study reported that other virulence genes, such as *peg-344* (putative transporter gene), *iroB* (salmochelin biosynthetic gene), *iucA* (iron-acquisition systems aerobactin), *rmpA*, and *rmpA2* (LuxR-like transcriptional regulators), contribute to hypervirulence [15].

Although cases of hvKp infection have been increasing since they were first reported in Taiwan in the mid-1980s [12], there is limited information on prostatic abscesses caused by *K. pneumoniae*. Further, the clinical significance of hvKp-related K1 or K2 capsular types or virulence genes in prostatic abscesses is still unknown. Therefore, the aim of this study was to elucidate the clinical and microbiological characteristics of prostatic abscesses caused by *K. pneumoniae* in relation to various virulence genes.

## 2. Materials and Methods

### 2.1. Patients and Data Collection

This single-center retrospective study was conducted in a 1200-bed tertiary hospital between January 2014 and December 2019. Patients diagnosed with a prostatic abscess with *K. pneumoniae* isolated from blood, urine, pus, or tissue cultures were enrolled. The diagnosis of prostatic abscess was confirmed using abdominopelvic computed tomography. hvKp is defined as when both *rmpA* and *iutA* genes are positive [14]. Demographic and clinical information were retrospectively collected from the patients’ electronic medical records, including age, comorbidities, clinical symptoms and signs, clinical outcomes, laboratory data, and treatment.

### 2.2. Microbiological Data and Antimicrobial Susceptibility

All laboratory tests were performed during routine clinical practice. The BacT/Alert 3D system (bioMérieux, Durham, NC, USA) was used for blood cultures, and all samples were inoculated into blood and MacConkey agar plates in a 5% CO_2_ incubator from 16 to 24 h at 35 °C. The microorganisms were identified using the VitekMS system (BioMérieux, Hazelwood, MI, USA), and antimicrobial susceptibility tests were conducted using Vitek2 AST 211 cards (BioMérieux, Marcy-l’Étoile, France) and interpreted using the VITEK2 identification systems [16]. We performed multiplex PCR to detect K1 and K2 capsular serotypes and virulence genes. Primer sets for *magA* (*wzy*-like polymerase specific to K1 capsular serotype), *wzi* (the gene specifying the K2 capsular serotype), and other virulence genes (*ybtS*, *entB*, *allS*, *kfu*, *iutA*, *mrkD*, and *rmpA*) have been described previously [17].

## 3. Results

Over the study period, 33 patients were suspected of having a prostatic abscess. Among them, 12 patients with prostatic abscesses caused by *K.* *pneumoniae* were identified. Two patients who were transferred from other hospitals for surgery were excluded due to a lack of clinical data, resulting in a final total of 10 patients included in the study.

The median patient age was 61 years (interquartile range, 54–75 years). Fever occurred in all patients, and dysuria or abdominal pain was the second most common symptom (50.0%). A total of nine (90.0%) patients had comorbidities, including five (50.0%) with diabetes mellitus (DM) and four (40.0%) with malignancies. Nine (90.0%) of the ten patients had community-acquired infections, while the other (10.0%) had a hospital-acquired infection. Eight patients (80.0%) had bacteremia. At the time of diagnosis, six (60.0%) patients had multiple sites of involvement, and the kidney (30.0%) was the second most common site of involvement after the prostate. The majority of patients recovered, while only one (10.0%) died, and there was no recurrence among the recovered patients. Laboratory data revealed pyuria in all patients. In addition, elevated prostate-specific antigen (PSA) levels were noted in five of the seven patients at the time of admission. The demographic and clinical characteristics of the 10 enrolled patients are summarized in Table 1.

In this study, the K1 serotype was detected in all patients with prostatic abscesses. Additionally, isolates recovered from eight patients (80.0%) carried the *rmpA* and *iutA* genes that identified hvKp (Table 2). In the antimicrobial susceptibility tests, eight *K. pneumoniae* strains were susceptible to all the tested antibiotics and two were extended-spectrum beta-lactamase (ESBL)-producing strains. Among the hvKp isolates, one was an ESBL-producing isolate, and all harbored virulence genes (*ybtS*, *entB*, *allS*, *kfu*, *iutA*, *mrkD*, and *rmpA*) excepting only one strain lacking *ybtS*. Details are given in Table 3.

## 4. Discussion

In this study, 30.3% (10/33) of the prostatic abscesses were caused by *K. pneumoniae.* All strains from patients with prostatic abscesses caused by *K. pneumoniae* carried the K1 capsular type, and 80.0% (8/10) were classified as hvKp. This study suggests that *K. pneumoniae*, especially hvKp, is an important pathogen of prostatic abscesses. This is the first study focusing on the K1/K2 capsular types and virulence factors of *K. pneumoniae* that cause prostatic abscesses.

In this study, *K. pneumoniae* (36.4%) was the leading pathogen causing prostatic abscesses, followed by *E. coli* (21.2%). This result is similar to a previous report from Taiwan [9]. However, this is inconsistent with a previous report from South Korea [10].

Until recently, there was a lack of consensus on a clear definition of hvKp. Defined as a combination of clinical and bacterial phenotypic features, hvKp has the ability to metastasize and is capable of causing invasive infection even in apparently healthy hosts [12]. The positive string test does not cover all mucoid colonies of *K. pneumoniae*, indicating a distinct difference between mucoid capsular strains and hypermucoviscous variants [18]. Hagiya et al. reported that non-hypermucoviscous strains can also be highly virulent when the microorganism possesses virulent genes such as *rmpA* or *magA* [18]. The hypervirulence phenomenon appears to be the consequence of complex interactions between multiple genetic determinants rather than a single gene [14,19]. A recent study demonstrated that *iroB, iucA, peg-344, rmpA*, and *rmpA2* are highly predictive molecular markers for hvKp [15]. Based on experimental data, *iuc, rmpA*, and *rmpA2* are the best-characterized virulence factors to date. The functions of *rmpA* and *rmpA2* may be redundant; therefore, *iuc* and/or either *rmpA* or *rmpA2* would be predicted to be best associated with the hvKp strain [14]. Based on the knowledge of previous studies, our study shows that 8 of the 10 strains carried hvKp and were the causative agents of prostatic abscesses. Although a limited number of cases were analyzed, this finding suggests that hvKp is more responsible for prostatic abscesses than cKp.

Invasive community-acquired infections can also be caused by hvKp, even in healthy adults, and such cases often involve multiple sites [12]. Our results show that all but one case of prostatic abscesses caused by hvKp were community-acquired infections. Of eight patients with hvKp, seven (87.5%) had at least one comorbidity, including DM (*n* = 5 (62.5%)), alcoholic liver cirrhosis (*n* = 2 (25.0%)), and malignancies (*n* = 2 (25.0%)). These results are consistent with those of previous studies [9,20,21]. Indeed, bacteremia, multiorgan abscess formation, and metastatic spread are well-known characteristics of hvKp [12]. Through this study, we affirmed that 75.0% of the patients with hvKp strains (6/8) had bacteremia and 62.5% (5/8) had multiorgan involvement, including one with central nervous system involvement. Among the organs involved, the kidneys were the most commonly affected (37.5%). However, renal abscesses are more common in women than in men [22]. Therefore, considering that the patients in this study are male, it is suggested that male patients with renal abscesses accompanied by bacteremia should be evaluated for prostatitis or prostatic abscesses.

Bacteria of the genus *Klebsiella* are notorious for their multidrug resistance, including carbapenemase-producing strains. Moreover, extended-spectrum beta-lactamase-producing *Klebsiella* are increasing. Fortunately, most hvKp strains are susceptible to most antimicrobial agents [11]. In this study, we observed that the majority of hvKp strains (7/8 (87.5%)) were susceptible to most antimicrobial agents tested. Nevertheless, the reported antimicrobial resistance in hvKp strains is increasing [23,24]. The carbapenemase-producing hvKp was first reported in the United States in 2019 [25]. Prior to this study, from June 2013 to September 2017 in China, 12.3% (13/106) of isolates were identified as carbapenemase-producing hvKp [26]. Recently, Jiang et al. reported a 5.6% positive rate for carbapenemase-producing hvKp from GenBank [27]. In our study, carbapenemase-producing hvKp was not found, but there was one extended-spectrum beta-lactamase (ESBL)-producing hvKp strain. Long-term use of antimicrobial agents has been widely accepted for the treatment of prostatic abscesses [28]. Consequently, ESBL- or carbapenemase-producing hvKp can limit the selection of antimicrobial agents available for prostatic abscesses and lead to treatment failure.

Although hvKp is endemic to the Pacific Rim and antimicrobial-resistant cKp is increasing in Western countries [14,29], the combination of antimicrobial-resistant cKp and hvKp seems to have already begun, and as mentioned above, there is evidence from recent studies suggesting that hvKp strains produce carbapenemase. Therefore, we believe that simultaneous monitoring of hvKp and antimicrobial resistance is necessary.

We revealed possible clinical characteristics of patients with prostatic abscesses caused by hvKp. However, this study has some limitations. First, salmochelin (iroA gene cluster, iroBCDEN) and *peg-344*, recently reported as significant virulence factors for hvKp [15], were not evaluated. Nonetheless, our experiments were performed with *iutA* and *rmpA*, the best-characterized virulence factors for hvKp. Second, as this was a single-center study of a small number of patients, the results should be interpreted with caution and cannot be generalized to all patients. Despite these limitations, our study has important clinical implications. In experiments focused on the K1/K2 capsular types and hypervirulent genes, we found that a substantial number of patients with prostatic abscesses caused by *K. pneumoniae* carried the hvKp strains.

In conclusion, our results suggest that hvKp is a significant pathogen in prostatic abscesses despite representing a rare disease in the post-antibiotic era. Therefore, when treating patients with prostatic abscesses caused by *K. pneumoniae,* caution should be exercised in checking for the characteristics of hvKp, such as bacteremia, multiorgan abscess formation, and metastatic spread.

## Figures and Tables

**Table 1:** Clinical characteristics of the patients with prostatic abscesses caused by *Klebsiella pneumoniae*.

	Case 1	Case 2	Case 3	Case 4	Case 5	Case 6	Case 7	Case 8	Case 9	Case 10
Age	60	79	54	41	62	75	82	58	71	45
Underlying disease	No	DM	DM	DM	Rectal cancer	Prostate cancer	Colon cancer	DM, alcoholic LC	DM, bladder cancer, s/p cystostomy	Heavy alcoholic
Type of infection	Community- acquired	Community-acquired	Community- acquired	Community- acquired	Community- acquired	Community- acquired	Hospital- acquired	Community- acquired	Community- acquired	Community- acquired
Tissue or Pus culture	KP	None	None	KP	KP	None	None	None	None	KP
Urine culture	None	KP	None	None	None	None	None	KP	KP	KP
Bacteremia	No	Yes	Yes	No	Yes	Yes	Yes	Yes	Yes	Yes
Other involved sites	Liver	No	Lung, brain, eye	No	Rt. thigh, gluteal area, and abdominal cavity	No	No	Kidney	Kidney	Kidney
Chief complaint	RUQ pain, Fever	Dysuria, fever	Visual disturbance, fever	Dysuria, fever	Abdominal pain, fever	Dysuria, fever	Fever	Fever	Fever	Fever
Antibiotics	Ceftriaxone IV, levofloxacin PO	Ceftriaxone IV, cefixime PO	Ceftriaxone IV, moxifloxacin PO	Ceftriaxone IV, levofloxacin IV, PO	Piperacillin– tazobactam, meropenem IV	Ceftriaxone IV, levofloxacin PO	Meropenem IV	Meropenem IV, TMP-SMX PO, levofloxacin PO	Meropenem IV, levofloxacin PO	Meropenem IV, ciprofloxacin IV, PO
Procedure or Operation	PCD (liver)	TURP	Vitrectomy	TURP	Graft removal	No	No	No	No	TURP
Outcome	Recover	Recover	Recover	Recover	Death	Recover	F/U loss	Recover	Recover	Recover
Sequalae	No	No	Blindness	Neurogenic bladder	No	No	No	No	No	No
Recurrence	No	No	No	No	N/A	No	N/A	No	No	No
Urinalysis (normal range)										
Protein (neg)	1+	1+	1+	2+	1+	2+	1+	2+	1+	Neg
RBC (1–4/HPF)	5–9	>30	1–4	26–30	0–2	>30	3–5	>30	>30	0–2
WBC (1–4/HPF)	>30	>30	>30	>30	3–5	>30	3–5	>30	>30	>30
Laboratory (scales)										
WBC (×10^3^/mm^3^)	19.90	10.49	17.20	13.46	15.66	12.55	19.49	2.60	17.18	27.38
Hb (g/dL)	13.00	7.60	12.10	12.30	8.40	10.70	15.40	9.50	10.10	9.80
Platelets (×10^3^/mm^3^)	358.00	116.00	242.00	279.00	438.00	170.00	102.00	44.00	434.00	116.00
ESR (mm/hr)	57.00	39.00	58.00	90.00	106.00	48.00	33.00	5.00	98.00	82.00
CRP (mg/L)	191.05	107.24	93.44	142.21	45.92	182.78	125.59	146.84	138.69	172.53
AST (IU/L)	59.00	33.00	39.00	16.00	17.00	38.00	167.00	117.00	15.00	162.00
ALT (IU/L)	109.00	16.00	52.00	18.00	23.00	17.00	164.00	30.00	16.00	119.00
BUN (mg/dL)	18.00	17.00	19.00	20.00	8.00	26.00	21.00	57.00	25.00	20.00
Creatinine (mg/dL)	0.70	0.79	0.79	0.61	0.52	2.02	1.03	2.11	1.40	1.36
PSA (0–4 ng/mL)	27.44	35.12	0.28	5.36	N/A	6.00	125.41	N/A	N/A	0.97

ALT, alanine aminotransferase; AST, aspartate aminotransferase; BUN, blood urea nitrogen; CRP, C-reactive protein; DM, diabetes mellitus; ESR, erythrocyte sedimentation rate; F/U, follow-up; Hb, hemoglobin; HPF, high-power field; IV, intravenous; KP, *K. pneumoniae;* LC, liver cirrhosis; N/A, not available; PCD, percutaneous catheter drainage; PO, per os; PSA, prostate-specific antigen; RBC, red blood cells; RUQ, right upper quadrant; TMP-SMX, trimethoprim–sulfamethoxazole; s/p, status post; TURP, transurethral radical prostatectomy; WBC, white blood cells.

**Table 2:** Virulence factors and serotypes of *Klebsiella pneumoniae* isolates in patients with prostatic abscesses.

	Case 1	Case 2	Case 3	Case 4	Case 5	Case 6	Case 7	Case 8	Case 9	Case 10
*rmpA*	+	+	+	+	-	+	-	+	+	+
*iutA*	+	+	+	+	-	+	-	+	+	+
*allS*	+	+	+	+	-	+	-	+	+	+
*entB*	+	+	+	+	+	+	+	+	+	+
*Kfu*	+	+	+	+	-	+	-	+	+	+
*mrkD*	+	+	+	+	+	+	+	+	+	+
*ybtS*	+	-	+	+	-	+	-	+	+	+
Serotype	K1	K1	K1	K1	K1	K1	K1	K1	K1	K1
ESBL	-	-	-	-	-	-	+	-	+	-

+, positive; -, negative; ESBL, extended-spectrum beta-lactamases.

**Table 3:** Antibiotic resistance in patients with prostatic abscesses caused by *Klebsiella pneumoniae*.

	Case 1	Case 2	Case 3	Case 4	Case 5	Case 6	Case 7	Case 8	Case 9	Case 10
Amikacin	S	S	S	S	S	S	S	S	S	S
Amp-sulb	S	S	S	S	S	S	R	S	R	S
Aztreonam	S	S	S	S	S	S	R	S	R	S
Ceftazidime	S	S	S	S	S	S	R	S	R	S
Cefazolin	S	S	S	S	S	S	R	S	R	S
Gentamicin	S	S	S	S	S	S	R	S	S	S
TMP-SMX	S	S	S	S	S	S	R	S	S	S
Pip-tazo	S	S	S	S	S	S	I	S	S	S
Ertapenem	S	S	S	S	S	S	S	S	S	S
Meropenem	S	S	S	S	S	S	S	S	S	S
Cefepime	S	S	S	S	S	S	R	S	R	S
Cefotaxime	S	S	S	S	S	S	R	S	R	S
Cefoxitin	S	S	S	S	S	S	S	S	S	S
Levofloxacin	S	S	S	S	S	S	R	S	S	S
Tigecycline	S	S	S	S	S	S	R	S	S	S

Amp-sulb, ampicillin–sulbactam; I, indeterminate; Pip-tazo, piperacillin–tazobactam; R, resistant; S, susceptible; TMP-SMX, trimethoprim–sulfamethoxazole.

## Data Availability

The data presented in this study are available on request from the corresponding author.
